# Serum proteomic profiling identifies transforming growth factor β receptor as a marker of persistent symptoms up to 18 months after mild SARS-CoV-2 infection

**DOI:** 10.1016/j.bbih.2026.101396

**Published:** 2026-10-22

**Authors:** Thor Ueland, Rebecca Jane Cox, Annika E. Michelsen, Elisabeth Berg Fjelltveit, Kuan Yang, Anders Bonnevie, Tuva Dahl, Fan Zhou, Pål Aukrust, Bjørn Blomberg, Kari Otterdal, Bente Halvorsen, Nina Langeland

**Affiliations:** a Research Institute for Internal Medicine, Oslo University Hospital Rikshospitalet, Norway; b Institute of Clinical Medicine, University of Oslo, Norway; c Section of Clinical Immunology and Infectious Diseases, Oslo University Hospital Rikshospitalet, Oslo, Norway; d Influenza Centre, University of Bergen, Norway; e Department of Clinical Science, University of Bergen, Norway; f Department of Microbiology, Haukeland University Hospital, Norway; g National Advisory Unit for Tropical Infectious Diseases, Haukeland University Hospital, Bergen, Norway; h Norwegian Institute of Public Health, Oslo, Norway

**Keywords:** COVID-19, SARS-CoV-2, Post-COVID condition, Transforming growth factor β receptor 2, Immunity

## Abstract

Persistent symptoms following the acute phase of SARS-CoV-2 infection have emerged as an increasing health problem. The pathogenesis for the development of post-COVID Condition (PCC) is still elusive. Herein we included serum samples from non-hospitalized (n = 189) and hospitalized (n = 52) COVID-19 patients, representing approximately 90% of all patients with confirmed SARS-CoV-2 infection in the municipality of Bergen, Norway between 28 February and 4 April 2020. The patients were followed clinically with systematic interviews for up to 18 months. Accompanying serum samples were analyzed by un-targeted proteomics and enzyme immunoassay (EIA). Our major findings were: (i) In a subpopulation of the total cohort (n = 69) proteome analyses identified 28 proteins that were dysregulated in individuals with mild and moderate PCC. Pathway analyses suggested the involvement of cell adhesion, extracellular matrix remodeling and immunity. (ii) The most significant finding were elevated serum levels of transforming growth factor β receptor 2 (TGFBR2) that were significantly higher in those with moderate as compared with those with mild and those without any PCC symptoms. (iii) This finding was conformed in the total cohort as assessed by EIA. (iv) The strongest association for TGFBR2 was found for impaired cognition that was seen at both 6, 12 and 18 months after infection. (v) In contrast to the home-isolated patients, no association of TGFBR2 to PCC symptoms were seen for those that were hospitalized during the acute infection. Our finding may indicate the involvement of TGFBR2 related pathways in the pathogenesis of PCC, in particular in those with cognitive symptoms.

## Highlights

• Proteomics show altered immunity and extracellular matrix in post-covid condition.
• Raised serum levels TGFBR2 were associated with the degree of symptoms.
• Persistently elevated TGFBR2 were particularly seen in those with cognitive symptoms.
• The relation of TGFBR2 to symptoms were not seen in those with severe acute disease.

## 1 Introduction

After the COVID-19 pandemic, persistent symptoms following the acute phase of SARS-CoV-2 infection have emerged as an increasing health problem for affected patients and their relatives, for the health care system and for society. The cumulative global incidence of post-COVID Condition (PCC), also called long COVID, is around 400 million individuals, which is estimated to have an annual economic impact of approximately $1 trillion, equivalent to about 1% of the global economy (Al-Aly et al., 2024). The symptoms in patients who develop PCC include fatigue, dyspnea and cognitive symptoms that vary in intensity and length (Blomberg et al., 2022; Michelen et al., 2021) and importantly, symptoms and findings following acute COVID-19 could also indicate an increased cardiovascular risk (Halvorsen et al., 2023; Zuin et al., 2023). More recent studies suggest increased risk for new-onset dementia in older adults (Shan et al., 2024; Zhang et al., 2025).

Several non-exclusive and overlapping pathogenic pathways seem to be involved in the pathogenesis of PCC. These include persistent and non-resolving activation of both innate and adaptive immunity, interaction with gut microbiota disturbances, thrombo-inflammation, dysfunctional neurological signaling, persistent SARS-CoV-2 replication as well as reactivation of other viruses including herpesviruses such as Epstein-Barr virus and human herpes virus 6 (Cervia-Hasler et al., 2024; Davis et al., 2023; Greenhalgh et al., 2024; Haunhorst et al., 2022; Peluso and Deeks, 2024; Peluso et al., 2023; Su et al., 2023b). However, the pathogenesis of PCC is still incompletely understood. Furthermore, while recent large-scale proteomic investigations have utilized targeted panels to analyze specific biomarkers in mostly hospitalized cohorts (Liew et al., 2024; Woodruff et al., 2023), a weakness with several studies is lack of comparative data between PCC mechanisms in patients with severe disease during acute SARS-CoV-2 infection, and those who experience milder disease. Moreover, several studies lack longitudinal data on the degree and type of PCC and accompanying levels of biomarkers.

To further elucidate the pathogenic mechanisms in PCC, we used an initial untargeted proteome approach to screen serum samples taken 6 months after the acute SARS-CoV-2 infection for molecules that could be related to development of PCC in a subgroup of the total cohort (n = 69). Candidate targets were then validated by enzyme immunoassays (EIAs) in the whole cohort (n = 241), representing approximately 90% of all patients with confirmed SARS-CoV-2 infection in the municipality of Bergen, Norway between 28 February and 4 April 2020 (Blomberg et al., 2021; Fjelltveit et al., 2023). The cohort was dominated by patients with mild and home-isolated acute SARS-CoV-2 infection, but for comparison, also included patients that were hospitalized during the acute infection. The patients were followed clinically with systematic interviews for up to 18 months with accompanying serum samples.

## 2 Materials and methods

### 2.1 Study population

The study population has previously been described (Blomberg et al., 2021; Fjelltveit et al., 2023) and included SARS-CoV-2 RT–PCR-positive patients diagnosed at Bergen Municipality Emergency Clinic (Kuwelker et al., 2021), and those admitted to the two neighboring city hospitals: Haukeland University Hospital and Haraldsplass Deaconess Hospital. Briefly, as part of a prospective cohort study with long-term follow-up, we consecutively enrolled home-isolated patients diagnosed with COVID-19 in the period 28 February to 4 April 2020, during the first wave of the pandemic in Bergen, Norway. For hospitalized patients, we accepted longer inclusion due to delay in hospitalization, up to 6 May. We included 189 home-isolated participants with available samples at 2, 6 and 12 months, and 143 had an additional sample at 18-month follow-up. The study population of home-isolated participants and 52 hospitalized patients with available blood samples is described in Table 1. Blood samples and questionnaires were collected at several time-points up to 18 months of follow-up.

**Table 1:** Demographic and clinical profile of COVID-19 cases.

Demographic and clinical profile of non-hospitalized COVID-19 positive cases in the discovery cohort				
	Asymptomatic (n = 34)	Mild (n = 16)	Moderate (n = 19)	*p*-value
Age	42 ± 12	40 ± 15	45 ± 12	0.57
Female sex	16 (47%)	6 (38%)	7 (37%)	0.71
BMI	24.3 ± 2.5	23.8 ± 3.3	26.3 ± 3.3	0.024
Comorbidities*	15 (44%)	7 (44%)	7 (37%)	0.86

Demographic and clinical profile of COVID-19 cases in the validation cohort.			
	Non-hospitalized (n = 189)	Hospitalized (n = 52)	*p*-value
Age	45 ± 16	56 ± 15	<0.001
Female sex	89 (47%)	29 (56%)	0.27
BMI	25.1 ± 3.3	27.9 ± 5.4	<0.001
Comorbidities*	79 (42%)	37 (71%)	<0.001
Total symptom prevalence at month 6/12/18	83 (44%)/87 (47%)/64 (49%)	40 (80%) at 6mo.	<0.001**
Available blood samples at month: 2/6/12/18	189/189/187/143	51/47/52/44	0.84

BMI, body mass index. *Comorbidities include asthma, chronic obstructive pulmonary disease, hypertension, chronic heart disease, rheumatic disease, diabetes, cancer, neurological disease, immunosuppressive conditions, or other severe or chronic disorders. ** comparing symptom prevalence at 6 months.

### 2.2 Ethical considerations

The study was approved by the Regional Ethics Committee of Western Norway (#118664). All eligible individuals received both oral and written information about the study protocol and provided written informed consent on inclusion.

### 2.3 Clinical data collection

Participant data was entered into electronic case report form (using the Research Electronic Data Capture Database REDCap, Vanderbilt University, Nashville, TN) software and subsequently stored on a secure research server. All cases recruited at Bergen Municipality Emergency Clinic were followed up for 18 months, with systematic interviews at 6, 12 and 18 months, and blood samples were collected at 2, 6, 12 and 18 months. The hospitalized patients were followed for a similar duration with blood sampling at each time-point; however, systematic interviews were only assessed at 6 months for this group for PCC. Comorbidities recorded and used for adjustment were the composite of asthma, chronic obstructive pulmonary disease, hypertension, chronic heart disease, rheumatic disease, diabetes, cancer, neurological disease, immunosuppressive conditions, or other severe or chronic disorders as previously reported (Fjelltveit et al., 2023). Symptoms related to fatigue and cognitive symptoms were recorded by the validated Chalder Fatigue Questionnaire (CFQ11) (Chalder et al., 1993) which is also validated in the Norwegian general population (Loge et al., 1998). PCC was defined as persistent or new onset symptoms 3 months after SARS-CoV-2 infection (Blomberg et al., 2021; Fjelltveit et al., 2023). The comparator control population was drawn from the home-isolated subgroup of our prospective cohort and designated as the asymptomatic control group. These individuals were confirmed COVID-19 cases who achieved full clinical recovery within 4 weeks of their acute illness and scored below the clinical thresholds required to be categorized with mild or moderate PCC during the follow-up assessments.

Selection of samples for the proteomic discovery was based on the CFQ11 with 34 samples from asymptomatic control group (CFQ11: 1-11) and 35 symptomatic patients (mild symptoms CFQ: 12-19; moderate symptoms CFQ11: 20-30). For mapping individual symptoms, Chalder Fatigue Questionnaire (CFQ-11) question 1 was used to score fatigue, while question 8 (impaired concentration) and question 11 (memory problems) were merged using a bimodal score to define cognitive symptoms. The severity of dyspnea was also recorded as a bimodal score.

### 2.4 Proteomics

Proteomics were analyzed using the Olink Explore HT platform (>5400 proteins; Olink Proteomics AB, Uppsala, Sweden) at the Olink Service Laboratory. Data preprocessing and statistical analyses of the log2-transformed Normalized Protein eXpression (NPX) values were performed in R (v4.1.0) using the OlinkAnalyze R package (v1.2.7). Differential protein expression between the symptom groups was evaluated via Olink_t-test. Proteins were considered significantly dysregulated if they met a Benjamini-Hochberg false discovery rate (FDR)-adjusted P-value < 0.05.

### 2.5 Blood sampling protocol and measurements of TGFBR2

Serum samples were separated and aliquoted before storage at −80^°^C. Serum levels of TGFBR2 were measured in duplicate by enzyme-immunoassay (EIA) using commercially available antibodies (Cat#DY241, R&D Systems, Minneapolis, MN) in a 384-format using a combination of a SELMA pipetting robot (Analytik Jena AG, Jena, Germany) and a BioTek dispenser/washer (BioTek Instruments, Winooski, VT). Absorption was read at 450 nm by using an enzyme immunoassay plate reader (BioTek Instruments) with wavelength correction set to 540 nm. Samples from home isolated and hospitalized patients were run on the same 384-well plate; the intra- and inter-assay coefficient of variation for TGFBR2 was 6.2% and 7.3%, respectively.

### 2.6 Analyses of IgG against SARS-CoV-2 spike protein

Serum samples were analyzed in a two-step EIA for endpoint titers, firstly by antibody screening for the Wuhan receptor-binding domain (RBD), followed by Wuhan spike EIA using the Wuhan spike protein, 100ng/well as previously described (Kuwelker et al., 2021; Trieu et al., 2021).

### 2.7 The microneutralization assay

The microneutralization assay was performed in a certified Biosafety Level −3 Laboratory using a local SARS-CoV-2 isolate from March 2020, hCoV-19/Norway/Bergen-01/2020 (GISAID accession ID EPI_ISL_541970) as previously described (Kuwelker et al., 2021; Trieu et al., 2021).

### 2.8 Statistical and bioinformatic analyses

Demographics were compared using T-tests and chi-square.

Proteomic data were assessed using one-way ANOVA with post-hoc analysis between groups which consisted of asymptomatic individuals and those with mild or moderate symptoms as determined by CFQ11. Proteins that were different between asymptomatic individuals and those with mild or moderate symptoms in False Discovery Rate (FDR) adjusted analysis were entered into a simple STRING analysis (https://string-db.org/) and functional enrichment was assessed. Further analysis was performed on proteins that were different between individuals with mild and moderate symptoms. These were visualized as a volcano plot and further tested by GLM analysis adjusting for age, sex and BMI.

The association between TGFBR2, determined by proteomics, and EIA was assessed with Pearson correlation. The temporal trajectory of TGFBR2 according to severity during acute COVID (i.e., hospitalized vs. home isolated) was analyzed by linear mixed models using untransformed levels and a log link function. The subject was used as random effect and severity and time as fixed effects (also as interaction) and age, sex and comorbidities as covariates.

The ability of TGFBR2 determined at 6-8 weeks to predict symptoms at 6-, 12- and 18-months follow-up, were assessed by Receiver Operating Characteristic (ROC) analysis and for selected symptoms also visualized as precision-recall curves. The average cut-off determined by Youden's index was used to dichotomize TGBR2, and odds of future symptoms was further assessed by logistic regression adjusting for age, sex and comorbidities. A model with log10 normalized TGFBR2 was also made and used to further assess odds in subgroups based on age, sex and comorbidities.

Serum levels of TGFBR2 (log10 transformed) were compared at each time-point between symptom groups in home-isolated cases using GLM with TGFBR2 as dependent, symptom as fixed factor and age, sex and comorbidity as covariates. Spearman's Rank-Order Correlation was used to assess correlations between TGFBR2 and IgG spike levels and microneutralization titers. Finally, TGFBR2 at 6-month follow-up relation to composite scores of persisting symptoms was assessed by Generalized Linear Model (GLM). P-values are two-sided and considered significant when <0.05.

## 3 Results

### 3.1 Demographic and clinical characteristics of the study cohort

Demographic data and clinical characteristics from discovery (proteome analyses) and validation (EIA analyses) cohorts are given in Table 1. For the discovery cohort assessed with O-link proteomics, demographics were similar between the different Chalder Fatigue score groups. In the entire cohort, those who had been hospitalized (i.e., severe disease, n = 52) were older, had more comorbidities and more symptoms compared to home-isolated cases (n = 189). All hospitalized patients were discharged within the recruitment period.

### 3.2 Proteomic discovery in relation to persisting symptoms at 6-month follow-up

We first performed proteomic discovery using the O-link Explore HT platform on serum samples from home-isolated COVID-19 individuals with persisting symptoms at 6 months according to predefined subgroups as determined by CFQ11. An overview of the selection and study layout showing the workflow from discovery to validation is given in Fig. 1A. As shown in Fig. 1B, we found 28 proteins that were dysregulated in individuals with mild and moderate persisting symptoms. All but two (5-azacytidine induced 2 [AZI2] and Cold-inducible RNA-binding protein [CIRBP]), both involved in stress responses and autophagy with potential protective effects on the host (Long et al., 2017; Ujevic et al., 2024; Zhu et al., 2024), displayed higher levels compared to asymptomatic patients in FDR adjusted analysis. Further analysis to distinguish those with mild and moderate symptoms revealed 3 proteins as shown in the volcano plot in Fig. 1C including AZI2, Cellular communication network factor 3 (CCN3) and TGFBR2, all among others involved in extracellular matrix (ECM) and immunity (Horn et al., 2022; Peng et al., 2021; Yeo et al., 2024). Finally, analysis by GLM adjusting for age, sex and comorbidities revealed that only TGFBR2 was higher in those with moderate symptoms compared to both asymptomatic patients and those with mild symptoms (Fig. 1D) and was selected for further assessment with EIA in the entire population.

**Fig. 1:**
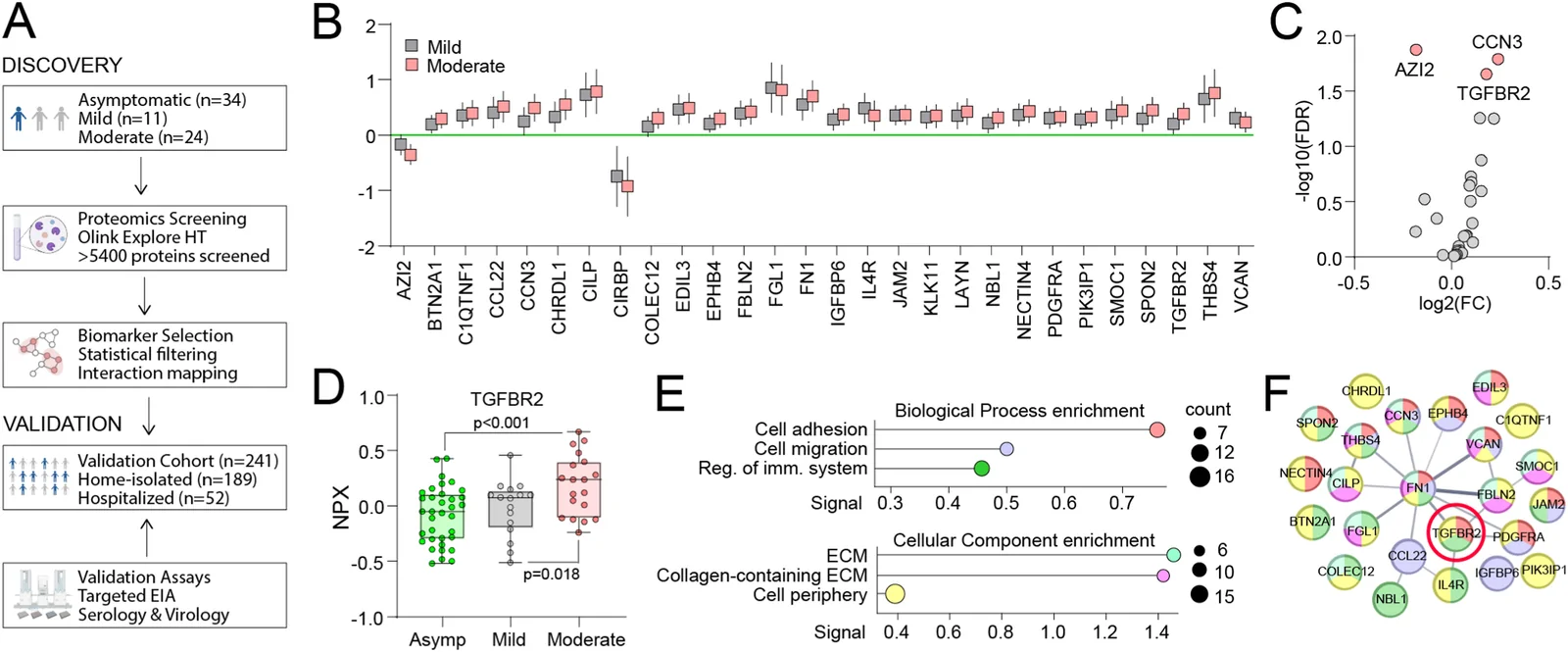
Dysregulated proteins in serum from home-isolated COVID-19 individuals with persisting symptoms at 6-month follow-up identified by the O-link Explore HT panel. **A)** Schematic overview of the study layout showing the workflow and patient distribution from the proteomic discovery subgroup (n = 69) to the full validation cohort (n = 241). Icons created with BioRender.com. **B)** 28 proteins dysregulated in individuals with mild and moderate persisting symptoms as determined by Chalder Fatigue Scale (CFQ11) compared to asymptomatic individuals in FDR adjusted analysis. Green line reflects the average in asymptomatic individuals. Definition of groups: CFQ11 scores <12: Asymptomatic; 12-19: Mild symptoms; 20-30: Moderate symptoms. **C)** Proteins distinguishing individuals with mild compared to moderate symptoms. **D)** Box and whiskers plot showing plasma levels of TGFBR2 determined by O-link in relation to persisting symptoms. P-values are adjusted for age, sex and comorbidities. **E)** STRING analysis of the 28 proteins identified in A showing enrichment for biological processes and cellular components. **F)** K-means clustering according to enrichment analysis. (For interpretation of the references to colour in this figure legend, the reader is referred to the Web version of this article.)

STRING analysis of the 28 proteins that were dysregulated in patients with persistent symptoms, showed biological process enrichment for cell adhesion and migration, regulation of the immune system, and cellular component enrichment for ECM and collagen-containing ECM and cell periphery (Fig. 1E–F), potentially suggesting a persistent, post-viral immunological condition involving remodeling of cellular surroundings that potentially could promote impairment of neurological function with effects on cognition.

### 3.3 TGFBR2 validation in the entire cohort and comparison with severe COVID-19 disease

As shown in Fig. 2A, comparison of TGFBR2 levels measured by EIA and O-link in the samples that were selected for proteomic analysis (n = 69) revealed a moderate correlation (r = 0.72, p < 0.001) between the methods. Measurements in the entire cohort at 4 time-points in home-isolated COVID-19 individuals (n = 189, mild SARS-CoV-2 infection) and those with severe COVID-19 disease (n = 52; hospitalized during acute diseases) demonstrated that levels in those with mild disease were stable throughout the follow-up period, while those with severe disease had higher levels up to 6 months followed by a decline reaching similar levels as those with mild disease at 18 months (Fig. 2B). Further, when evaluating TGFBR2 levels in relation to Chalder score subgroups at 6 months, as performed in the discovery cohort, there was a modest increase in levels with higher Chalder score, but with large overlap and marginal significance (p < 0.05) in those with mild and moderate symptoms compared to asymptomatic individuals (Fig. 2C). In contrast, no association with symptom scores was observed in individuals with severe acute disease (Fig. 2D). Finally, in both mild and severe acute COVID-19, TGFBR2 levels correlated modestly with IgG spike levels and microneutralization titers (Fig. 2E).

**Fig. 2:**
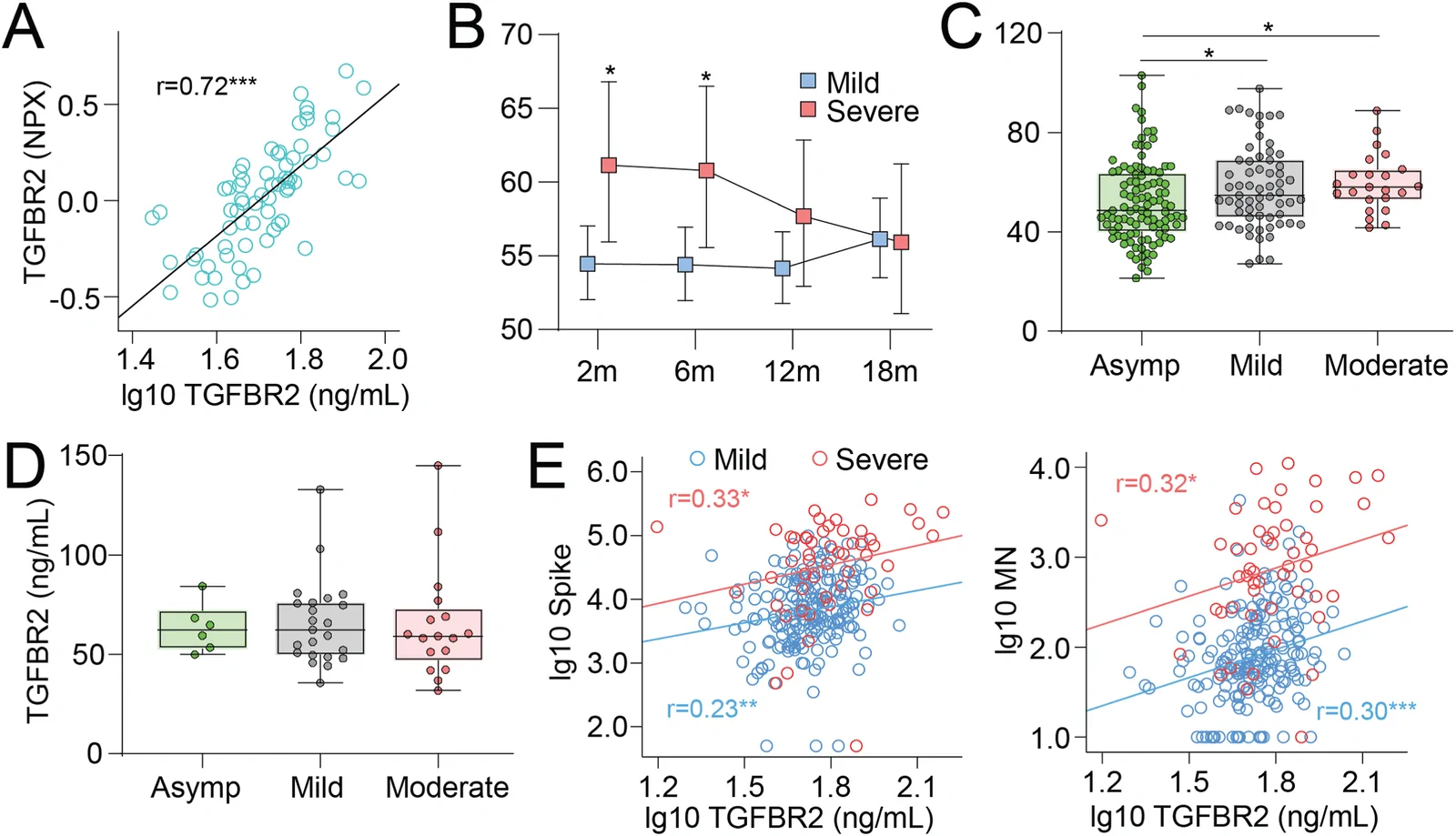
Validation of TGFBR2 in home-isolated COVID-19 individuals (mild disease) with persisting symptoms and comparison with severe COVID-19 disease (hospitalized patients) in the validation cohort. **A)** Correlation (Pearson) between TGFBR2 determined by O-link and enzyme-immunoassay (EIA). p < 0.001. **B)** Serum levels of TGFBR2 determined by EIA in the validation cohort during 18 months of follow-up following mild or severe COVID-19 disease. Data are shown as estimated marginal means with 95% CI. *p < 0.05 vs. mild disease. Box and whiskers plot showing plasma levels of TGFBR2 determined by EIA in relation to persisting symptoms at 6-month follow-up in **C)** mild and **D)** severe COVID-19 disease. P-values are adjusted for age, sex and comorbidities. Definition of groups: CFQ11 scores <12: Asymptomatic; 12-19: Mild symptoms; 20-30: Moderate symptoms. **E)** Association between TGFBR2 and IgG spike levels and microneutralization titers in mild and severe COVID-19 disease. *p < 0.05, **p < 0.01, ***p < 0.001.

### 3.4 TGFBR2 in home-isolated COVID-19 cases during long-term follow-up in relation to specific persisting symptoms

We next focused on TGFBR2 in relation to PCC symptoms in mild COVID-19 cases where serum and symptom scores were analyzed at the same time-point. There were relatively few individuals who scored as having moderate symptoms on the specific scales and symptoms were assessed categorically (i.e. yes/no).

As shown in Table 1, symptom prevalence was stable across the three time-points from 6 to 18 months with 44-49% reporting at least one or more symptoms. Likewise, the prevalence of specific symptoms in these home-isolated patients was relatively stable across these time-points as shown in Fig. 3A. The prevalence of dyspnea was 14-16%, fatigue 32-37% and cognitive symptoms were registered in 27-35% of individuals.

**Fig. 3:**
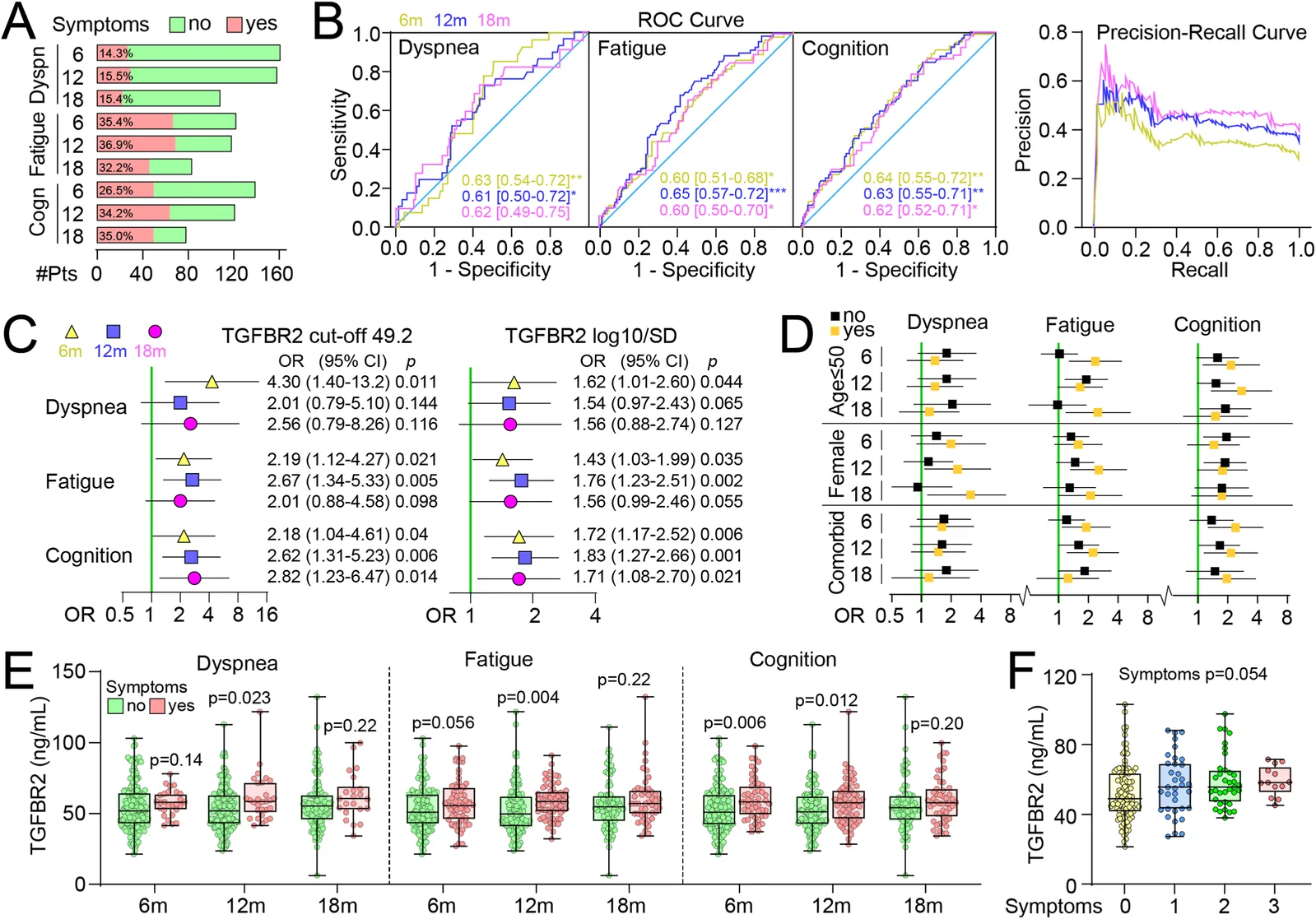
TGFBR2 i n home-isolated COVID-19 cases during long-term follow-up in relation to specific persisting symptoms of dyspnea, fatigue and cognition in the validation cohort (n = ). **A)** Prevalence of symptoms at 6, 12 and 18 months after infection. Data are shown superimposed and number of individuals is given on the x-axis while percentage with symptoms is noted on the bars. **B)** Receiver operating curve of plasma TGFBR2 determined at 6-8 weeks according to symptoms (yes/no) at 6, 12 and 18 months follow-up. The area under the curve is shown for each follow-up time-point. *p < 0.05, **p < 0.01 and ***p < 0.001. The right panel shows a Precision-Recall curve for TGFBR2 at 6-8 weeks in relation to cognitive symptoms at 6, 12 and 18 months. **C)** Logistic regression showing associations between TGFBR2 at 6-8 weeks and symptoms at 6, 12, and 18 months. In the left forest plot TGFBR2 levels were dichotomized according to Youden's index (average level from curves in B while the right shows odds according to log-normalized levels (i.e. log10/SD). Estimated marginal means and 95 CI's are given in the graph following adjustment for age, sex and BMI. **D)** Logistic regression showing associations between TGFBR2 at 6-8 weeks and symptoms at 6, 12, and 18 months according to age (above or below 50 years), sex and presence of comorbidities. Sex, age and comorbidities were included as covariates where appropriate. **E)** Tukey plots showing serum levels of TGFBR2 in relation to symptoms at 6, 12 and 18 months after infection (i.e. serum samples and symptoms at the same time-point). Data were analyzed by multivariate GLM adjusted for age, sex and comorbidities. **F)** TGFBR2 at 6-month follow-up relation to composite scores of persisting symptoms. Overall adjusted (age, sex, comorbidities) p-value for symptoms from GLM analysis is shown.

We first assessed the ability of TGFBR2 measured 6-8 weeks after infection in predicting specific symptoms during long-term follow-up. As shown in the ROC curves in Fig. 3B, TGFBR2 was associated with most symptoms at all time-points but with relatively poor discriminatory capability (AUC between 0.60 and 0.65). Further evaluation of the precision-recall curve suggested that a high recall could be achieved but that the majority (>50%) would be false positive limiting the use of TGFBR2 as a meaningful clinical biomarker for PCC.

Using a threshold of 49.2 (average Youden's index), we assessed if high levels of TGFBR2 (above threshold) at 6-8 weeks were associated with future symptoms in adjusted analysis. As shown in Fig. 3C, having high TGFBR2 was associated with 2-3 times higher odds of fatigue and cognitive symptoms in adjusted analysis (age, sex, comorbidities) and similar coefficients but larger confidential intervals for dyspnea, probably due to lower incidence. Similar associations were found when TGFBR2 was evaluated as a continuous log normalized variable (i.e., log10/SD). Thus, one SD increase in TGFBR2 was associated with 1.4-1.8 times higher odds of specific symptoms. Evaluation of normalized TGFBR2 and specific symptoms in relation to age, sex and comorbidities revealed no interaction between these subgroups and TGFBR2 levels (Fig. 3D).

Next, we investigated TGFBR2 levels measured at the same time as symptom assessment. As shown in Fig. 3E, serum TGFBR2 levels in relation to specific symptoms largely mirrored the findings above when evaluating the predictive ability of TGFBR2 at 6-8 weeks. Thus, a small but significant elevation in TGFBR2 was observed at 12- months follow-up in those with fatigue and in those with dyspnea and in 6- and 12-months in those with cognitive symptoms. Finally, we checked if TGFBR2 levels were associated with symptom burden as reflected by composite scores categorizing symptoms as having 0, 1, 2 or 3 of the selected symptom scales (i.e., dyspnea, fatigue, cognition). While a modest increase in levels was observed with increasing symptom burden, there was large overlap with an overall p = 0.054 (Fig. 3F).

## 4 Discussion

The pathogenic mechanisms leading to PCC are still not clear. We analyzed serum samples from approximately 90% of all patients with confirmed SARS-CoV-2 infection in the municipality of Bergen, Norway between 28 February and 4 April 2020, showing that serial serum levels of TGFBR2 up-to 18 months after the acute infection were independently associated with persistent PCC symptoms in cases with mild disease course, but not in hospitalized cases, with severe acute SARS-CoV-2 infection. These findings together with proteome analyses may suggest that molecular pathways related to ECM remodeling and immunity could be involved in the pathogenesis of PCC.

Previous studies suggest TGF-β signaling is involved in lung pathology may augment maladaptive pulmonary remodeling with development of lung fibrosis following acute SARS-CoV-2 infection (Yao et al., 2023). Herein, we show the association of serum levels of TGFBR2 to PCC was not restricted to those with persistent dyspnea as a potential sign of pulmonary pathology but was more closely associated with fatigue and cognitive symptoms (i.e., concentration and memory impairment).

The role of TGF-β signaling in the brain is dual, with beneficial and detrimental effects depending on context. Thus, TGFBR2 signaling supports synaptic plasticity and is crucial for maintaining microglial homeostasis as well as blood-brain barrier integrity through effect on endothelial cells (Hellbach et al., 2014; Luo, 2022; Senatorov et al., 2019; Su et al., 2023a). Conversely, activation through TGFBR2 may also cause excessive ECM remodeling and fibrosis in neural tissues following vascular injury and neuroinflammation (Kim et al., 2017; Zode et al., 2011). In the context of cognition, inhibition of TGFBR2 has been shown to improve cognition in pre-clinical models (El Hamamy et al., 2025) while a lower expression of TGBR2 and impaired TGF-β signaling is associated with cognitive decline in neurodegenerative disease (Fessel, 2020; Tesseur et al., 2006). However, as soluble TGFBR2 may act as a decoy receptor, binding TGF-β ligands in the extracellular environment and reducing downstream signaling (Rowland-Goldsmith et al., 2001), further studies are needed to determine if increased circulating TGFBR2 reflects attenuated or augmented TGF-β signaling and if this is related to cognition in the context of PCC. Finally, and with some relevance to PCC, serum levels of TGFβ, including TGFβ2, are elevated in subgroups of patients with Myalgic Encephalomyelitis/Chronic Fatigue Syndrome, related to circadian rhythm disruption (McCarthy, 2022), a phenomenon common in PCC (Livieratos et al., 2025). Moreover, although the proteome analyses should be interpreted with caution, the downregulation of AZI2 and CIRBP and upregulation of CCN3 could also be related to disturbed ECM remodeling and immunity as well as to increased sensitivity to various stress responses (Horn et al., 2022; Long et al., 2017; Peng et al., 2021; Ujevic et al., 2024; Yeo et al., 2024; Zhu et al., 2024) of which the latter has been linked to PCC (Yavropoulou et al., 2022).

While serum levels TGFBR2 were associated with PCC symptoms in those with mild acute SARS-CoV-2 infection, this was not seen in those who were hospitalized during the acute infection. This latter group also had a different pattern during follow-up with high levels of TGFBR2 at 6 months followed by a decline reaching similar levels to individuals with mild disease at 18 months. These findings underscore that in hospitalized patients, the disease severity *per se* may be a risk factor for PCC (Ueland et al., 2025; Xie et al., 2021), and some caution is needed when interpreting data in relation to PCC from hospitalized patients, that may not reflect the pathogenic mechanisms in non-hospitalized patients and vice versa. This clinical distinction is highlighted by comparing our results to large-scale proteomic investigations of long-COVID. Studies such as the post-hospitalization cohort by Liew et al. (2024), the longitudinal IMPACC study (Gabernet et al., 2025), and the cohort analyzed by Woodruff et al. (2023) primarily focused on individual clinical traits or targeted protein arrays for pre-selected markers. While these studies emphasize generalized innate immune activation, metabolic shifts, or systemic autoreactivity, our approach focused on TGFBR2 and included validation with an alternative methodology (immunoassay) in a larger population. Furthermore, the association between TGFBR2 and cognitive scores is consistent with observations by Liew et al. (2024), where specific symptom profiles mapped to individual molecular alterations in plasma. The divergence in protein findings across different studies notes that variations in patient populations and testing methods can yield different molecular profiles. We have previously shown that elevated spike IgG and microneutralization titers are associated with the occurrence of PCC symptoms in this cohort (Fjelltveit et al., 2023). Herein we show that serum levels of TGFBR2 levels correlated modestly with IgG spike levels and microneutralization titers. The reason for this pattern is not clear, whether it reflects an association between TGFBR2 levels and high viral load during the acute phase or a potential “side effect” of the immune response to SARS-CoV-2.

The present study has some limitations. The cohort was infected with the Wuhan strain, and our findings could not necessarily be applied to later SARS-CoV-2 variants such as various omicron strains. Moreover, all the patients were unvaccinated, and the role of SARS-CoV-2 vaccination in relation to PCC development could therefore not be examined. Moreover, although validated questionnaires were used, we lack data on objective organ function such radiological examination, pulmonary testing and neuro testing. In addition, correlations do not necessarily imply any causal relationship, and more mechanistic studies are needed to further explore the role of TGFBR2 in PCC. Furthermore, the diverse findings observed across the current proteomic literature highlight the complexity of post-COVID pathology and underscore the necessity for independent validation studies using objective clinical measurements. Strengths of our study include longitudinal data on symptom scores as well as longitudinal serum samples from up-to 18 months after the acute infection. The study included a near-complete geographical cohort from the first pandemic wave and a personalized follow-up to detect long COVID symptoms, which may be missed in healthcare-based registry studies. The inclusion of both hospitalized and non-hospitalized patients is also a strength of the study.

The present study shows an association between serum levels of TGFBR2 and PCC symptomatology in patients who had been home-isolated for mild acute SARS-CoV-2 infection, but not in those hospitalized for severe COVID-19. Our finding does not suggest that serum levels of TGFBR2 represent a biomarker of PCC but may indicate the involvement of TGFBR2 related pathways in the pathogenesis of PCC, in particular in those with cognitive symptoms.

## CRediT authorship contribution statement

**Thor Ueland:** Conceptualization, Data curation, Formal analysis, Investigation, Methodology, Visualization, Writing – original draft, Writing – review & editing. **Rebecca Jane Cox:** Conceptualization, Data curation, Investigation, Methodology, Writing – review & editing. **Annika E. Michelsen:** Data curation, Methodology, Writing – review & editing. **Elisabeth Berg Fjelltveit:** Data curation, Investigation, Writing – review & editing. **Kuan Yang:** Data curation, Formal analysis, Investigation, Methodology, Writing – review & editing. **Anders Bonnevie:** Data curation, Investigation, Writing – review & editing. **Tuva Dahl:** Data curation, Formal analysis, Investigation, Methodology, Writing – review & editing. **Fan Zhou:** Data curation, Investigation, Writing – review & editing. **Pål Aukrust:** Conceptualization, Formal analysis, Investigation, Writing – original draft, Writing – review & editing. **Bjørn Blomberg:** Conceptualization, Data curation, Investigation, Methodology, Writing – review & editing. **Kari Otterdal:** Conceptualization, Formal analysis, Funding acquisition, Writing – review & editing. **Bente Halvorsen:** Conceptualization, Formal analysis, Funding acquisition, Project administration, Writing – review & editing. **Nina Langeland:** Conceptualization, Formal analysis, Investigation, Methodology, Project administration, Writing – review & editing.

## Ethics declaration

Written informed consent to take part in the study and to publish the article has been obtained from all participants or their legal representatives. The privacy rights of participants have been observed.

This study included organ or tissue donors. This study includes human biological material and consent was obtained by donors, or their next of kin or legal representatives, for use in this study and for publication of the article. The samples used in this research were not sourced from executed prisoners or prisoners of conscience.

This study was performed in compliance with relevant laws, regulatory frameworks and guidelines where the research took place. This study was approved by the South-Eastern Norway Regional Health Authority. (Approval No. 2021071 and 2022063)

## Declaration of competing interest

The authors declare that they have no known competing financial interests or personal relationships that could have appeared to influence the work reported in this paper.
